# Ureter Rupture From Ureterovesical Junction Obstruction in a Child

**DOI:** 10.7759/cureus.83933

**Published:** 2025-05-11

**Authors:** Chen-Yu Chang, Yu-Wei Fu, Yi-Jung Chen, Shao-Yen Wu

**Affiliations:** 1 Pediatric Nephrology, Changhua Christian Hospital, Changhua, TWN; 2 Pediatric Surgery, Changhua Christian Hospital, Changhua, TWN

**Keywords:** case report, congenital urinary obstruction, ureterovesical junction obstruction, ureter rupture, urinary ascites

## Abstract

Ureteral rupture in children with ureterovesical junction obstruction (UVJO) is rare. We report the case of a five-year-old boy diagnosed with right UVJO who was lost to follow-up for one year and later referred to our department, presenting with acute abdominal pain. An abdominal computed tomography (CT) revealed right hydronephrosis, hydroureter, and unilateral ascites. Serum creatinine level significantly decreased within six hours, suggesting urinary ascites due to ureteral rupture. Dimercaptosuccinic acid (DMSA) kidney scintigraphy demonstrated significantly decreased function of the right kidney (16%) compared to the left (84%). Pediatric surgery revealed right UVJO with a ruptured distal right ureter, which was reconstructed surgically. As this case illustrates, routine follow-up of patients with congenital urinary tract obstruction is critical to avoid extreme morbidity.

## Introduction

Ureterovesical junction obstruction (UVJO) is a congenital anomaly that obstructs the normal flow of urine from the ureter into the bladder, potentially leading to hydronephrosis, urinary tract infections, or progressive renal damage [1]. UVJO is the second leading cause of hydronephrosis in newborns following ureteropelvic junction obstruction (UPJO), responsible for about 20% of cases [2]. Although UVJO is a recognized cause of obstructive uropathy, spontaneous ureteral rupture secondary to UVJO is extremely rare, particularly in children. Most reported cases of ureteral rupture are associated with trauma, iatrogenic injury, or stones; cases associated with congenital anomalies have been seldom reported [3].

Due to its nonspecific clinical manifestations, ureteral rupture poses a diagnostic challenge, as it often mimics other causes of acute abdomen. Early recognition and appropriate imaging are essential to prevent irreversible renal damage. In cases of urinary ascites resulting from rupture, a rapid decline in serum creatinine may serve as a useful biochemical clue for diagnosis [4].

We report a case of spontaneous ureteral rupture in a five-year-old boy with a previously diagnosed right-sided UVJO who had been lost to follow-up for one year. This case underscores the importance of close clinical monitoring and timely intervention in patients with congenital urinary tract obstruction.

## Case presentation

**Figure 1:**
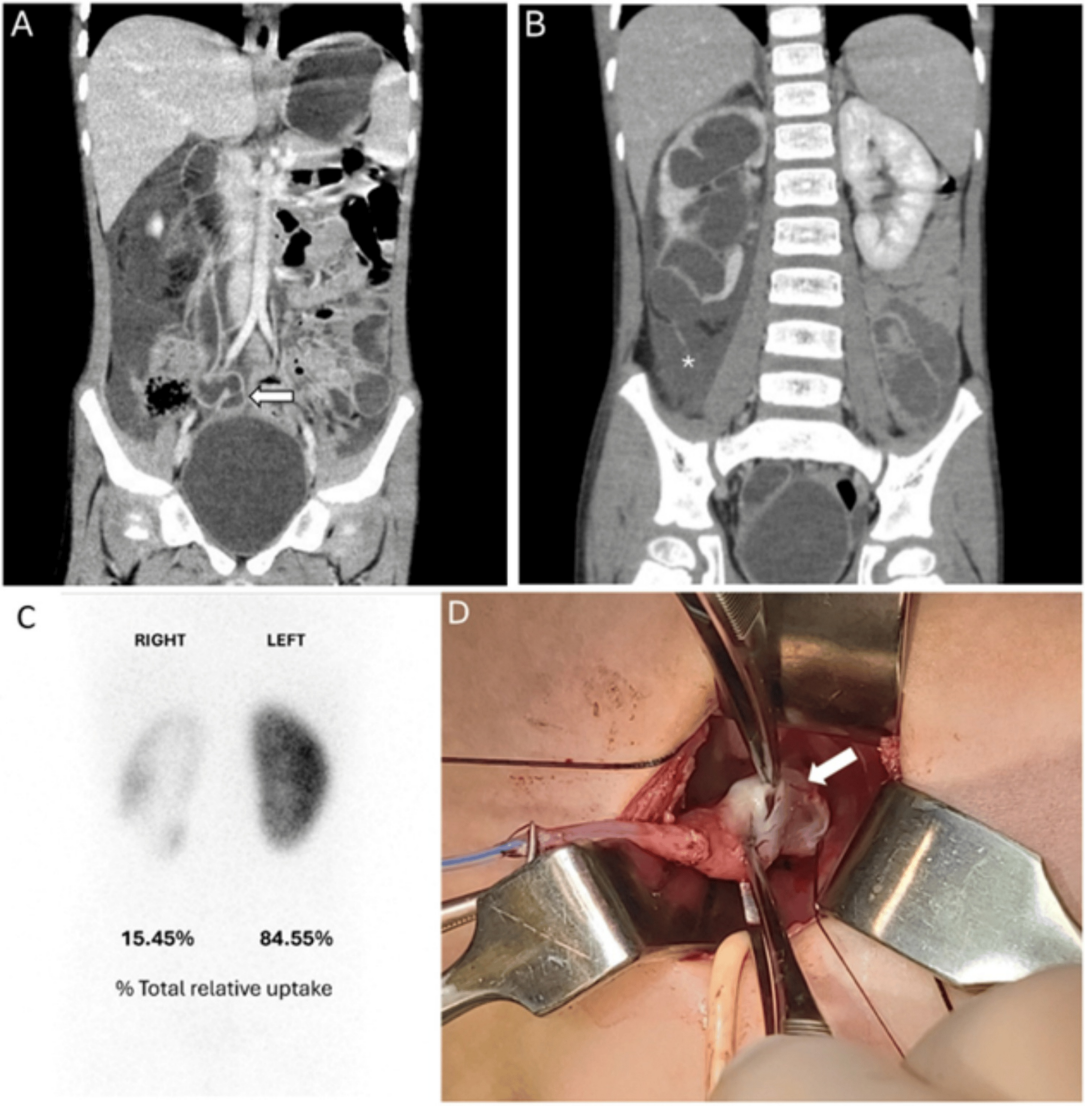
Imaging and Intraoperative Evidence of Our Case (A) Abdominal computed tomography showing significant right-sided hydronephrosis and hydroureter (arrow) (B) Abdominal computed tomography showing ascites localized to the right side (asterisk) (C) Dimercaptosuccinic acid kidney scintigraphy demonstrating severely impaired right kidney function (16%) and preserved left kidney function (84%) (D) Intraoperative image showing rupture in the distal right ureter (arrow)

This is a five-year-old boy with no known congenital malformations at birth. He was previously diagnosed with right UVJO, resulting in mild right hydroureteronephrosis. The last two ultrasounds of his kidneys, two months apart, were virtually unchanged. He was lost to follow-up for a year before presenting to the emergency department with acute, severe abdominal pain. At home, he was lying on the floor, screaming for help, but did not show any external injuries. Abdominal computed tomography (CT) revealed marked right-sided hydronephrosis and hydroureter (Figure 1A, arrow) without evidence of stones and right-sided ascites (Figure 1B, asterisk). Dimercaptosuccinic acid (DMSA) kidney scintigraphy showed severely impaired function in the right kidney (16%) and preserved function in the left kidney (84%) (Figure 1C). Voiding cystourethrography (VCUG) revealed no evidence of vesicoureteral reflux (VUR) in either ureter.

Laboratory workup showed a serum creatinine of 1.12 mg/dL (normal range: 0.25-0.53 mg/dL [5]) that quickly decreased to 0.3 mg/dL in six hours. This finding strongly suggested that urinary ascites had been reabsorbed into the circulation, transiently elevating serum creatinine levels. The left kidney compensated for the impaired function of the right kidney due to hydroureteronephrosis, by effectively eliminating excess creatinine.

Two days later, repeat ultrasonography confirmed the stabilization of hydronephrosis and resolution of ascites. The surgery performed one week later revealed a bladder wall stricture and a distal rupture of the right ureter (Figure 1D, arrow). The ureter was reconstructed surgically. The patient had an uneventful postoperative course and continued to have the right kidney closely monitored for recovery of function.

## Discussion

Ureteral rupture in children with UVJO is rare. The cause of spontaneous ureteral rupture reported in the literature is typically associated with stones [6]. This case represents an unusual presentation in their absence. Other differential diagnoses should be considered, including metastatic invasion of the ureter, urinary retention secondary to retroperitoneal fibrosis [7], pregnancy [8], neurogenic bladder, connective tissue disease [9,10], and ureteral strictures. Causes include previous surgery, radiation therapy [11], autoimmune disease, or tumor [6].

Acute abdominal or flank pain remains the most common presenting symptom in patients with ureteral rupture [3,12]. This sharp, often debilitating pain typically prompts urgent medical evaluation. In children, however, symptom expression may be less specific, leading to delayed diagnosis. Early recognition is essential, as it enables timely imaging studies such as ultrasound or contrast-enhanced CT to identify urinoma formation, retroperitoneal fluid collections, or direct signs of rupture and its underlying etiology [13,14]. In our case, abdominal CT initially revealed massive ascites and hydroureteronephrosis, raising suspicion of urinary tract perforation.

UVJO is a congenital defect in which urine does not flow from the ureter to the bladder, resulting in progressive hydroureteronephrosis [2]. Without proper regular follow-up, the obstruction can progress silently and eventually present serious complications including rupture, urinary ascites, and kidney injury as seen in this case.

Principally, an initial rise in serum creatinine may suggest acute kidney injury. However, in this patient, a rapid decline was likely due to peritoneal absorption of urinary ascites [4], as creatinine levels in acute kidney injury typically remain elevated for days. In this case, DMSA kidney scintigraphy showing impaired kidney function in the affected kidney directed the surgical planning. Thereafter, early surgical intervention was performed to prevent irreversible kidney damage and to facilitate long-term kidney function preservation.

This case highlights the importance of routine follow-up in pediatric patients with congenital urinary tract obstruction. As the disease is often asymptomatic, continued aggressive monitoring allows earlier detection of progression, providing an opportunity for intervention to prevent serious complications such as kidney injury and spontaneous rupture, which carry tremendous morbidity.

## Conclusions

This rare case of spontaneous ureteral rupture in a child with UVJO highlights the severe consequences of delayed recognition in congenital urinary tract anomalies. Routine follow-up and timely intervention are essential to reduce the risk and preserve kidney function.
